# From the American Journal of Medical Sciences.—Case of Exostosis of the Upper Jaw

**Published:** 1839-12

**Authors:** B. A. Rodrigues

**Affiliations:** Charleston


*From the American Journal of Medical Sciences.—Case of Exostosis of the upper jaw, by*

[ B. A. RODRIGUES, M. D. OF CHARLESTON. ]

On the 14th of August, 1837, Charity, a servant woman of Mrs. Miller, called on me to ascertain whether I could afford her any relief in her wretched condition. She had been laboring under incessant and agonizing pain in the antrum highmorianum of the right side, which she regarded as the consequence of the impaired condition of the teeth. On this supposition, she had several of them extracted, without any appreciable abatement of her sufferings. Yet deluded with the belief that some one of the remaining teeth was the secret agent of all she suffered, she persisted in having more extracted. Still the evil continued, the suffering was unabated, the cause undetected; and to add to the depression of her hopes, and the aggravation of her ills, a purulent discharge oozed from the empty sockets of the affected side. She again had recourse to medical advice, hoping that this new phasis of her malady, might lead to some indication that would relieve her; at least, that it might reveal its hidden sources, its condition, and its prospects of being remediable. And here for the first time, was it suggested that the antrum was in an unsound state.

It was at this moment, under these circumstances, that she applied to me to perform an operation, which her medical adviser declared to be indispensable. At first, I imagined it to be an abscess of the cavity from the pus discharged, from the strange sensations experienced, and from the greater frequency of this disease over others peculiar to this part. I inserted a trochar into the socket of the second molar, and instead of the gush of matter I had expected, the passage of the instrument was intercepted by a hard, dense, impregnable substance. The existence of an exostosis now forced itself on me. To make assurance doubly sure, I had access to several of my medical friends, among whom, was Dr. Geddings. On examination of the part, the consideration of the symptoms, the obstinate nature of the disease, they concurred with me in opinion, that an exostosis was present, and that the sole indication of relief was its extirpation. Accordingly, on the 18th of August, the above gentleman, with several others of the profession was present, when I proceeded to perform the operation. With a common scalpel I dissected away the gum from the canine teeth to the last molar, raised the flap which it made from the alveolar process, and with a trephine opened into the cavity. Success was easier than had been anticipated in consequence of the carious condition of the process which was so general on the affected side as to reach from the second incisor anteriorly to the pterygoid process posteriorly. In the loss of substance, the external parietes of the cavity shared, so that the bony tumour which filled up and occupied it, could be readily reached. The trephine was applied, the cavity enlarged, and the exostosis removed. It measured in circumference three inches, was light, and cancellated on its surface, but dense and resisting in its more internal layers. There was little or no hemorrhage to delay the operation, or any application to arrest it. After removing every spiculum of diseased bone, and cleansing out the cavity, the flap was replaced and to nature was entrusted the cure. Granulations operated up in full luxuriance, and in the short period of four weeks, the woman was in the enjoyment of excellent health. It may be well to remark, that when I saw her for the first time, the only outward symptom that the disease presented that might have determined the diagnosis of exostosis, was the occlusion of the nasal cavity. Respiration through this natural channel was impossible, but such an obstacle, I can readily conceive, may occur from a high and acute inflammation in the living membrane of the part. An incipient abcess is almost invariably announced by such an obstruction, and a preponderance of the affection over the other naturally suggested its existence. Baudernaue and Abernethy have both noticed the presence of exostosis in these cavities, but to my recollection there has been no history of them recorded, where the tumour was so large, where such extensive injury was inflicted on the adjacent parts, and where nature, after the cause of malady had been removed, exerted her recuperative powers so benignly and so quickly. Its early history, its duration, its probable causes, whether local or constitutional, are involved in mystery, the patient calling on me but a short time before the operation, and seeming to know nothing more of it than her sufferings.

The foregoing, is one of the most remarkable and interesting cases of exostosis of the antrum highmorianum on record; and it is to be regretted, that more of the early history of the case than is given in the report, could not be obtained. As it is, we are left altogether to conjecture as to the causes which gave rise to the disease. It is very likely, that, could all the circumstances connected with its whole history have been ascertained, its origin might have been traced to a diseased condition of some one or more of the teeth of the affected side. BLT. ED.

